# Survey of the Condition of Each Tooth in Patients With Mandibular Protrusion by Age Group

**DOI:** 10.7759/cureus.80930

**Published:** 2025-03-21

**Authors:** Ko Nakanishi, Mami Mutoh, Kaoru Matsumura, Machiko Kasai, Ryoshun Endo, Emi Kikuchi, Shuhei Otsubo, Kazuhiro Matsushita, Takaaki Yamamoto, Yoshiaki Sato

**Affiliations:** 1 Department of Biomaterials and Bioengineering, Faculty of Dental Medicine, Hokkaido University, Sapporo, JPN; 2 Department of Orthodontics, Faculty of Dental Medicine and Graduate School of Dental Medicine, Hokkaido University, Sapporo, JPN; 3 Stomatognathic Function, Center for Advanced Oral Medicine, Hokkaido University Hospital, Sapporo, JPN

**Keywords:** caries, dmft, jaw anomaly, mandibular protrusion, missing tooth

## Abstract

Background: Jaw deformities are known to cause occlusal collapse. However, the process of occlusal collapse is not clear. This study evaluated the dental health status of each tooth in patients with mandibular protrusion by age group to determine which teeth are affected by mandibular protrusion and clarify the progression of occlusal collapse.

Methods: This study evaluated the condition of each tooth in 113 patients with mandibular protrusion at the Orthodontic Department of Hokkaido University Hospital from 2013 to 2017 by age group. Patients were examined for decayed, missing, filled, and root canal-treated teeth. Filled teeth were classified into teeth with filling composite resin and inlay (Filled tooth), teeth with attached crown (Crown tooth), and abutment teeth of bridge by treatment methods.

Results: The Decayed, Missing, and Filled Teeth (DMFT) index for mandibular protrusion patients for 10s, 20s, 30s, 40s, 50s, and 60s was 5.3, 10.2, 14.8, 19.3, 19.8, and 18.7, respectively, which was higher in all age groups than in the results of the 2016 Survey of Dental Diseases in Japan. The survival rates of the lower molar tended to be low. The Filled tooth rate tended to be higher for molars than for other teeth for all ages. The Crown and abutment tooth rate increased in various teeth in the maxilla. In the mandible, the Crown tooth rate in the premolar and molar greatly increased, and the abutment tooth wasn't found in the incisor. The root canal-treated tooth rate was higher in the molar with increasing age in the mandible.

Conclusion: The findings raised the possibility that patients with mandibular protrusion may be more susceptible to some oral diseases, such as caries, periodontal disease, and occlusal trauma, and the condition of the molar region may tend to deteriorate compared to other regions.

## Introduction

Malocclusion is a condition caused by various factors [1,2], such as genetics and habit. It affects oral function (speech, mastication, and deglutition), esthetics, and mental health [3]. Therefore, the World Health Organization (WHO) regards malocclusion as one of the most important oral health problems following dental caries and periodontal disease [4]. Especially, jaw deformities with severe skeletal anterior-posterior or lateral discrepancies between the maxilla and mandible pose a higher burden to patients [5]. Orthodontic treatment with surgery is required for treating jaw deformities. The number of patients receiving treatment has recently increased due to growing concerns for oral function or esthetics, resulting in patient satisfaction with treatment outcomes, such as chewing function and facial appearance [6].

One of the known disadvantages of malocclusion is the increased incidence of dental caries [2,7,8] and periodontal disease [9-11]. The relationship between malocclusion and dental caries has been discussed for decades [12]. In 1988, Addy et al. [13] reported a significant increase in plaque in irregular teeth, but no significant differences in the incidence of dental caries. In 2004, Stahl and Grabowski [14] found no positive correlation between the prevalence of malocclusion and caries in children with primary dentition; however, they observed statistically significant parallelism in the prevalence of malocclusion and caries in children with mixed dentition. In 2019, Doğramacı and Brennan [2] reported that the long-term caries experience of participants orthodontically treated for malocclusion was slightly lower than that for non-orthodontically treated patients but with no significant differences. On the other hand, Oz and Kucukesmen [11] examined the relationship between malocclusion and periodontal disease and found that lower anterior segment crowding was detrimental to periodontal health. However, few reports have examined the relationship between malocclusion and caries or periodontal disease only in patients with jaw deformities. Therefore, this study focused on patients with jaw deformities, especially mandibular protrusion, and evaluated their relationship with dental disease.

Mandibular protrusion (skeletal Ⅲ malocclusion) caused by anterior-posterior discrepancies between the maxilla and mandible is one of the representative malocclusions. In severe cases requiring orthodontic surgery, patients almost have an anterior crossbite. Anterior crossbite prevents adequate anterior guidance, interferes with stable occlusion due to losing a proper occlusal stop [15], and could impose a burden on the molars during mastication. Moreover, most patients with anterior crossbite have labial tipping of maxillary anterior teeth and lingual tipping of mandibular anterior teeth as dental compensation [16]. It can also impose a burden on anterior teeth. Surgical orthodontic treatment aims to prevent occlusal collapse resulting from these burdens. However, few reports have evaluated the progression of occlusal collapse in patients with mandibular protrusion.

This study evaluated the dental health status of each tooth in patients with mandibular protrusion by age group to determine which teeth are affected by mandibular protrusion and clarify the progression of occlusal collapse.

## Materials and methods

Study participants

Among the 188 patients with jaw deformities requiring orthognathic surgery for orthodontic treatment at the Orthodontic Department of Hokkaido University Hospital from January 2013 to December 2017, this study included 113 patients diagnosed with mandibular protrusion (Table 1). Patients with congenital diseases, such as cleft lip and/or palate or other syndromes, were excluded from this study.

**Table 1:** The age distribution in this study

Age (years)	Number
15-19	36
20-29	40
30-39	14
40-49	15
50-59	6
60-69	2
Sum	113

Investigation of tooth condition

Patients were divided into 10s, 20s, 30s, 40s, 50s, and 60s and examined for decayed, missing, and filled teeth using panoramic radiographs, oral photographs, and medical records in the initial examination to calculate the Decayed, Missing, and Filled Teeth (DMFT) index, tooth survival rate for each tooth, and decayed tooth rate for each tooth. These items were calculated using the following formula: (a) DMFT index=the total number of DMF teeth/the number of patients, (b) tooth survival rate=the total number of teeth that have not been lost/the number of patients, and (c) the decayed tooth rate=the total number of decayed teeth/the number of patients×100.

In addition, this study examined the treatment methods for filled teeth in order to clarify the condition of the tooth. Filled teeth were classified into teeth with filling composite resin and inlay (Filled tooth), teeth with attached crown (Crown tooth), and abutment teeth of bridge. The rate of Filled tooth, Crown tooth, and abutment teeth was calculated for each tooth. The teeth that received root canal treatment were surveyed from panoramic radiographs, and the rate of teeth receiving root canal treatment was calculated for each tooth. These rates were calculated using the following formula: (a) the rate of Filled tooth=the number of Filled tooth/the number of patients×100, (b) the rate of Crown tooth=the number of Crown tooth/the number of patients×100, (c) the rate of abutment teeth=the number of abutment teeth/the number of patients×100, and (d) the rate of teeth that received root canal treatment=the number of teeth that received root canal treatment/the number of teeth×100.

Ethical approval

This study was approved by the Ethics Committee of the Hokkaido University Hospital (approval number: JI017-0427). The Ethics Committee agreed that informed consent was not required according to the Ethics Guidelines for Medical Research Involving Human Subjects, as this study did not involve samples taken from the human body. Information about the conduct of the research, including the purpose of the research, was published, and all participants were given the opportunity to refuse to take part in the research.

## Results

DMFT index

**Figure 1:**
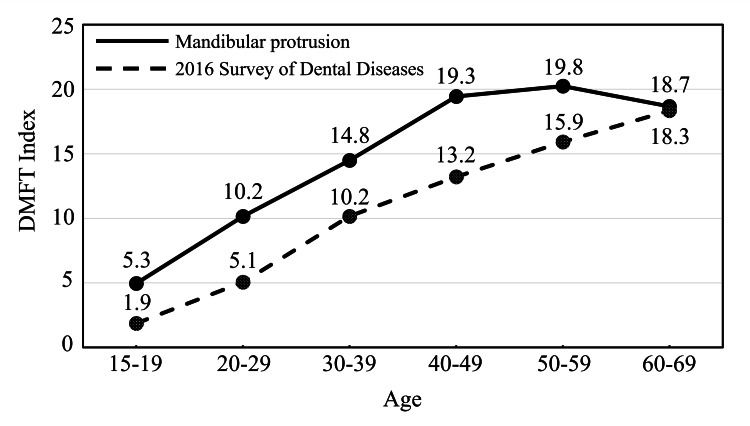
DMFT index DMFT: Decayed, Missing, and Filled Teeth

Figure 1 shows the results of the DMFT index. The 2016 Survey of Dental Diseases in Japan (the national survey in Japan), conducted every six years, was used as a control. The DMFT index for mandibular protrusion patients for 10s, 20s, 30s, 40s, 50s, and 60s was 5.3, 10.2, 14.8, 19.3, 19.8, and 18.7, respectively, which was higher in all age groups than in the results of the 2016 Survey of Dental Diseases in Japan.

Survival rate of each tooth

Table 2 and Table 3 show the survival rate of each tooth. The average included all patients from 10s to 60s. In the 10s, there were no missing teeth except for the lower right lateral incisor. After the 20s, various teeth were missing. In the upper arch, the canines had a slightly higher survival rate than other teeth, with no major trend in the survival rate of other teeth. In the lower arch, the survival rate of molars tended to decrease with increasing age after the 30s. The survival rate of incisors, canines, and first premolar was high for all ages.

**Table 2:** The survival rate of each upper tooth

		Upper tooth													
		17	16	15	14	13	12	11	21	22	23	24	25	26	27
Age (years)	15-19	1.00	1.00	1.00	1.00	1.00	1.00	1.00	1.00	1.00	1.00	1.00	1.00	1.00	1.00
	20-29	1.00	0.93	0.97	1.00	0.98	1.00	1.00	0.98	1.00	1.00	1.00	0.95	0.98	0.95
	30-39	1.00	0.93	0.93	0.86	1.00	0.92	0.86	0.93	1.00	1.00	0.93	0.86	0.86	0.93
	40-49	0.93	0.93	0.73	0.87	0.93	0.80	0.93	0.93	0.93	1.00	0.93	0.87	0.80	1.00
	50-59	0.67	1.00	0.50	0.67	1.00	0.83	1.00	1.00	0.50	1.00	1.00	1.00	0.83	0.67
	60-69	0.50	0.50	0.50	1.00	1.00	0.50	0.50	1.00	1.00	1.00	1.00	0.50	0.50	1.00
	Average	0.96	0.95	0.91	0.95	0.98	0.95	0.96	0.97	0.96	1.00	0.98	0.95	0.93	0.96

**Table 3:** The survival rate of each lower tooth

		Lower tooth													
		47	46	45	44	43	42	41	31	32	33	34	35	36	37
Age (years)	15-19	1.00	1.00	1.00	1.00	1.00	0.97	1.00	1.00	1.00	1.00	1.00	1.00	1.00	1.00
	20-29	1.00	1.00	0.98	1.00	1.00	1.00	1.00	1.00	1.00	1.00	1.00	1.00	0.98	0.98
	30-39	0.93	0.79	1.00	1.00	1.00	1.00	1.00	1.00	1.00	1.00	1.00	0.93	0.86	0.86
	40-49	0.93	0.87	0.87	1.00	1.00	1.00	1.00	1.00	0.93	1.00	0.93	0.87	0.80	0.60
	50-59	0.50	0.67	0.83	1.00	1.00	0.83	1.00	1.00	1.00	1.00	1.00	1.00	0.50	0.67
	60-69	0.00	0.00	0.50	1.00	1.00	1.00	1.00	1.00	1.00	1.00	0.50	0.50	0.00	0.00
	Average	0.93	0.92	0.96	1.00	1.00	0.98	1.00	1.00	0.99	1.00	0.98	0.96	0.90	0.88

The decayed tooth rate of each tooth

**Figure 2:**
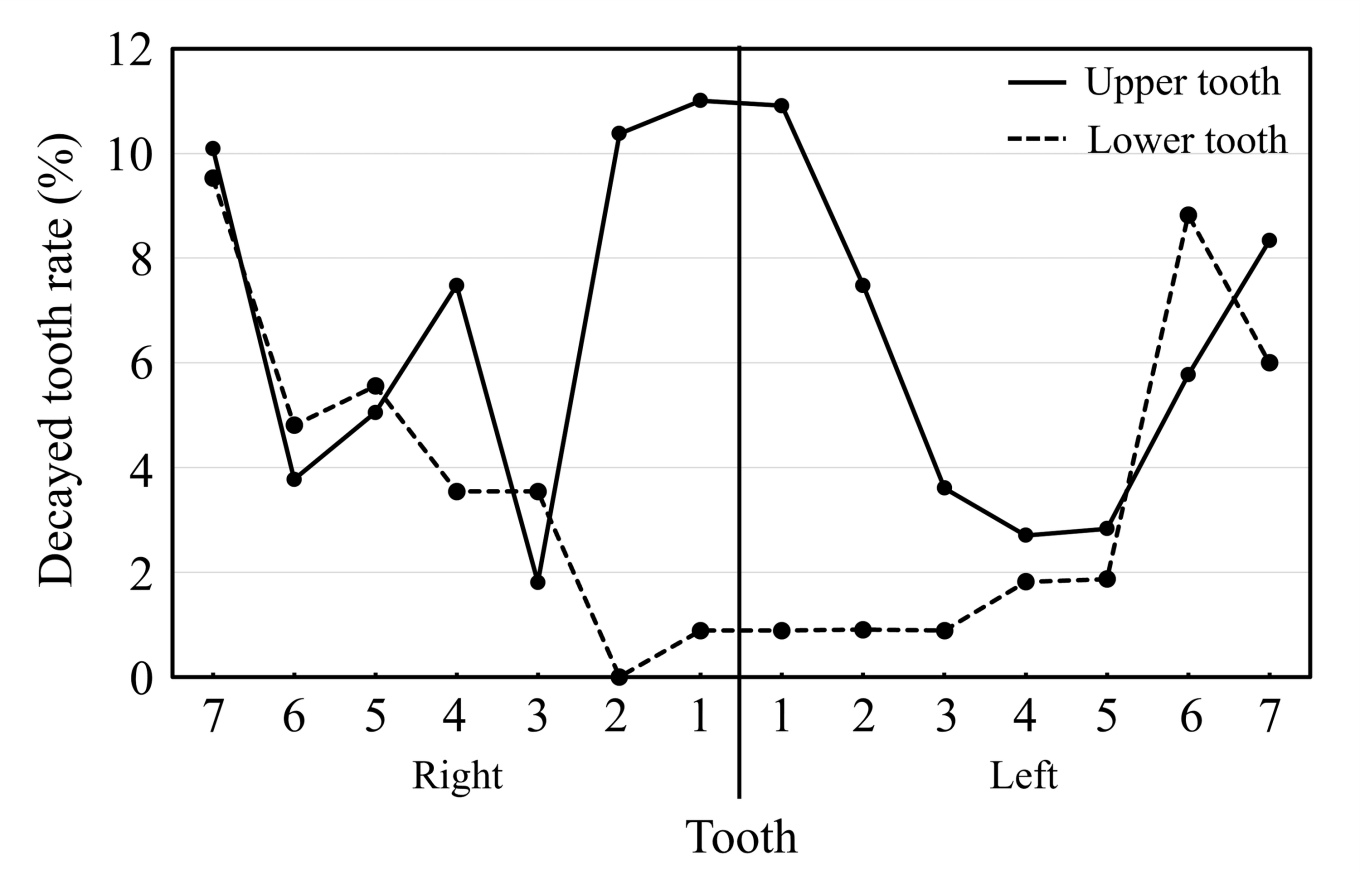
Decayed tooth rate

Figure 2 shows the decayed tooth rate for each tooth for all patients. The decayed tooth rate of the upper incisors tended to be higher, and the upper right central incisor had the highest decayed tooth rate at 11%, followed by the upper and lower second molar; the lower incisors tended to show the lowest decayed tooth rate.

Tooth condition rate

**Figure 3:**
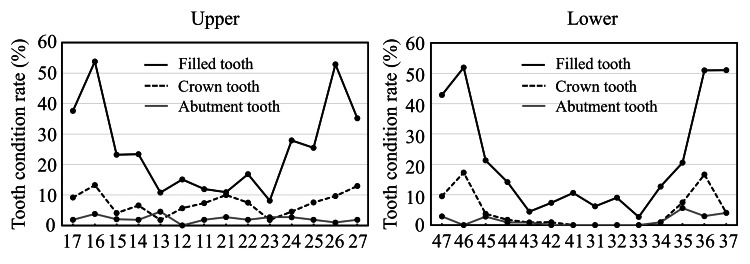
Tooth condition rate

Figure 3 shows the tooth condition rate for all patients. Filled teeth were most frequent in molars (both maxillary and mandibular). The Crown tooth rate in the maxilla was lower in canines. In the mandible, crown teeth were less frequent in incisors, canines, and first premolars. The abutment tooth rate was lower in both the maxilla and the mandible, with no abutment in the lower incisor.

**Figure 4:**
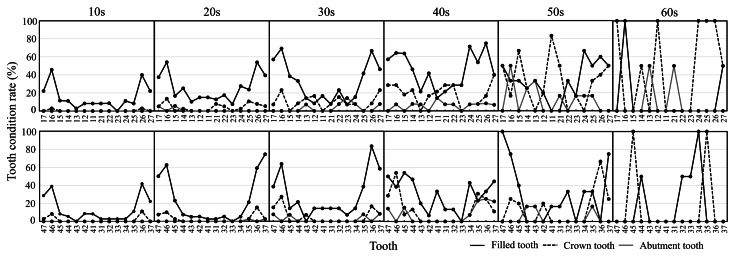
Tooth condition rate by age group

Figure 4 shows the tooth condition rate by age group. The Filled tooth rate tended to be higher in molars than in other teeth for all ages. In addition, in the 40s, the Filled tooth rate increased, except for molars. The only Crown tooth in the 10s was the first molar in the maxilla. From the 20s, the Crown tooth rate also gradually increased in other upper teeth. The mandibular arch showed the Crown tooth only in the molar in the 10s and the premolar in the 20s. After the 30s, the Crown tooth rate in the premolar and molar showed a notable increase. There was no Crown tooth in the lower incisor until the 40s. The abutment teeth were present in the maxilla and mandible in the 10s. The abutment tooth rate increased in various teeth from the 20s in the maxilla. However, in the mandibular arch, the abutment teeth were not present in the incisor until the 60s. There was no tendency in the 50s and 60s due to the small number of survey participants.

Root canal-treated teeth rate

**Figure 5:**
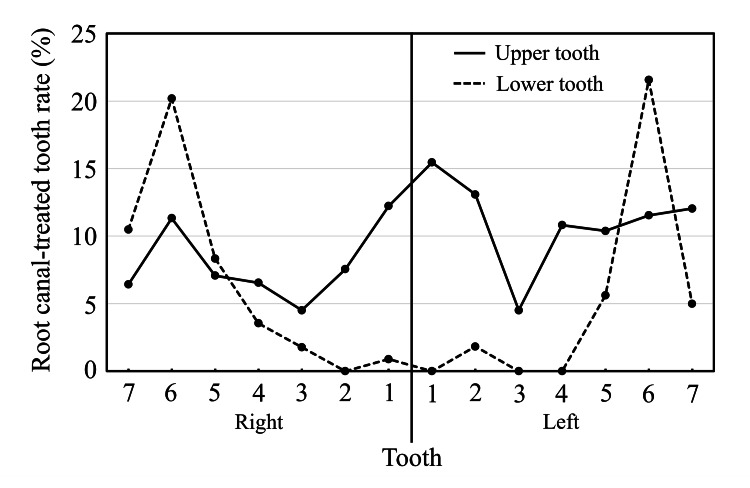
Root canal-treated tooth rate

Figure 5 shows the root canal-treated tooth rate for all patients. This rate was high for the maxillary incisors and mandibular molars.

**Figure 6:**
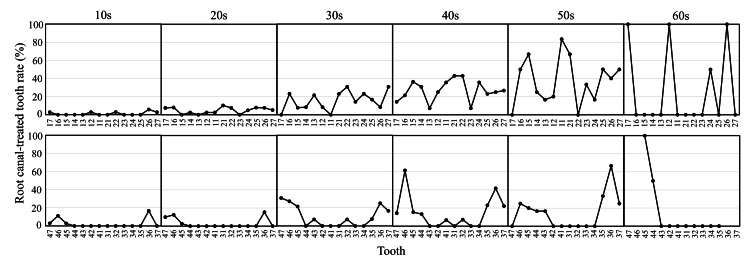
Root canal-treated tooth rate by age group

Figure 6 shows the root canal-treated tooth rate by age group. In the maxilla, this rate gradually increased in all teeth with age. In the mandibular arch, this rate showed a marked increase in molars with increasing age compared to other teeth. There was no tendency in the 50s and 60s due to the small number of survey participants.

## Discussion

This study evaluated patients with jaw deformities (severe malocclusion) to clarify the relationship between malocclusion and dental disease. Almost all patients with jaw deformities have remarkable characteristics of malocclusion in tooth inclination and mastication, regardless of the condition, compared to patients with malocclusion who do not require surgery for treatment, because they have large skeletal discrepancies. Therefore, it is suitable to select patients with jaw deformities as targets for clarifying the relationship between malocclusion and dental disease. This study used patients with jaw deformities with mandibular protrusion and investigated each tooth condition in each age group to evaluate the relationship between mandibular protrusion and dental disease.

The prevalence of Angle Ⅰ malocclusion, Angle Ⅱ malocclusion, and Angle Ⅲ malocclusion in permanent dentition was 55.5%, 24.7%, and 10.7%, respectively, according to Lombardo et al. [17] and 74.7%, 19.56%, and 5.93%, according to Alhammadi et al. [18]. However, in recent years, among the patients surgically treated for jaw deformities, there have been slightly more patients with mandibular protrusion than those with maxillary protrusion [19,20]. Venugoplan et al. [19] reported that the percentage of patients with maxillary hyperplasia or mandibular hypoplasia and mandibular hyperplasia or maxillary hypoplasia among all patients treated with surgical orthodontic treatment in 2008 was 47% and 30%, respectively. Proffit et al. [20] reported that the percentage of patients with maxillary protrusion and maxillary deficiency or mandibular protrusion among all patients treated with surgical orthodontic treatment at North Carolina University from 1996 to 2000 was 59% and 35% and from 2006 to 2010 was 41% and 54%, respectively. Proffit et al. [20] suggested two reasons for the increased rate of patients with maxillary deficiency or mandibular protrusion. One was that mandibular deficiency was more socially acceptable than mandibular excess or maxillary deficiency in a general population largely of European origin. The other reason was that the number of African American, Hispanic, Native American, and Asian patients having orthognathic surgery, which constitutes a higher percentage of maxillary deficiency or mandibular protrusion, increased. Thus, the rate of patients with mandibular protrusion requiring surgical orthodontic treatment has increased, and mandibular protrusion is attracting interest. Therefore, it is more important to clarify the disadvantage of leaving mandibular protrusion untreated by encouraging dentists to promote orthodontic treatment and persuading patients to undergo treatment.

The DMFT index for patients with mandibular protrusion was higher in all age groups than in the 2016 Survey of Dental Diseases results in Japan (Figure 1). This study is a cross-sectional study, not a longitudinal study. Therefore, the history of treatment or cause of tooth loss was unknown. The Filled tooth could have received treatment for caries or fractures due to occlusion or trauma, and tooth loss could have occurred due to caries or periodontal disease. Some patients with mandibular protrusion could have received upper anterior prostheses to correct the anterior crossbite. This study's results confirmed that patients with mandibular protrusion had a worse state of teeth. Patients with mandibular protrusion are more susceptible to oral diseases such as caries and periodontal disease. Although the DMFT index was higher in Hokkaido than in all of Japan, the results of the 2011 Survey of Dental Diseases in Hokkaido showed that the DMFT was 1-2 teeth higher in the 30s and above than the national average [21]. However, the DMFT index of patients with mandibular protrusion was still higher. Although regional characteristics of Hokkaido may have contributed to the results of this study, mandibular protrusion could have also affected the DMFT index.

This study investigated tooth condition by age group to clarify the reason for high DMFT index values in patients with mandibular protrusion and identify the teeth that got worse first. However, we found no major trends in the 50s and 60s due to the small number of survey participants.

The survival rate of the molar was slightly lower than other teeth after the 20s when the patients began to show tooth loss, implying that the loss rate of molars was slightly higher than that of other teeth. Molars also tended to have a relatively high rate of the Filled tooth, Crown tooth, and root canal-treated teeth, with the first molar showing high rates from an early age, suggesting that the condition of the first molar deteriorates earlier in patients with mandibular protrusion. Studies show that this tendency is also the same in normal occlusion. The first molars are susceptible to caries because they are exposed to the oral environment for longer than other teeth due to their early erupting period and have deep pits and fissures [22,23]. Moreover, patients with mandibular protrusion have very steep chewing angles and flatter occlusal guidance, and they masticate food mainly by chopping [24]. Because chopping is less effective than grinding, the masticatory performance of patients with mandibular protrusion is less than that of normal occlusion. Studies show that deficient mastication decreases self-cleaning function [25]. Therefore, patients with mandibular protrusion have lower self-cleaning function than those with normal occlusion, resulting in higher rates of missing teeth, filled teeth, crown teeth, and root canal-treated teeth. The low self-cleaning function may influence other teeth, including the first molar. Many patients with mandibular protrusion diagnosed with jaw deformity have an anterior crossbite or edge-to-edge bite, and almost all these patients don't show anterior guidance with anterior teeth but with posterior teeth, imposing a significant burden on the molars than normal occlusion. As a result, the condition of the molars is predicted to worsen.

This study showed a slightly higher rate of missing upper second premolars. The main occlusal area could be one of the reasons. The main occlusal areas are the upper first molar and lower first molar in patients with normal occlusion and the upper second premolar and lower first molar in mandibular protrusion [26]. We assume that the burden of the maxilla second premolar in patients with mandibular protrusion during chewing, which causes the fracture of the tooth and worsens marginal and periapical periodontal disease, is greater than that of other teeth.

Periodontal disease may also be involved in the rate of missing teeth, crown teeth, and root canal-treated teeth. Root canal treatment may be performed in case of severe periodontal disease which reaches the apex of a tooth followed by prosthetic treatment. In 1972, Geiger et al. [9] reported that Class III patients showed more gingival inflammation or gingival disease than patients with normal occlusion. In 2008, Bollen [10] reported that anterior crossbites caused gingival recession and tooth mobility, although he did not mention mandibular protrusion. These reports suggest that mandibular protrusion worsens periodontal disease. In this study, periodontal disease with mandibular protrusion may have raised the rate of missing teeth, crown teeth, and root canal-treated teeth. However, there are various opinions on the relationship between malocclusion and periodontal disease, necessitating further investigation.

It was found that the survival rate and the rate of decayed, filled, crown, abutment, and root canal-treated teeth were approximately the same for left and right homonymous teeth. The reason for the difference from this symmetry depending on partly age group was predicted to be few subjects. Another reason was possible mandibular lateral displacement. The occlusal guidance inclination in the buccal segment of the nonshifted side is steeper in patients with mandibular lateral displacement than in the shifted side [27]. We assume that this change in occlusal guidance inclination affected masticatory performance, related to the self-cleaning function mentioned above, and masticatory burden, related to tooth fracture and marginal and periapical periodontal disease. A survey of patients with mandibular lateral displacement is required to clarify the effect of displacement on tooth condition.

Tooth loss due to caries or periodontal disease affects surrounding teeth, and in many cases, it exacerbates the condition, such as aggravation of periodontal tissue, resulting in occlusal collapse. The molars, especially the first molars, have wide occlusal surfaces with the most effective oral masticatory ability [22]. Therefore, molars should remain caries-free to avoid tooth loss. The results of this study suggest that patients with mandibular protrusion are susceptible to oral diseases, including caries, especially in molars. The risk of oral diseases can be reduced by the early management of mandibular protrusion to prevent oral collapse. This is something that should be widely promoted to the public in order to protect oral health, and it can be used to provide evidence for recommending orthodontic treatment to patients with mandibular protrusion.

The limitation of the study is that it was only conducted in Hokkaido, Japan. The prevalence of oral diseases, including dental caries and periodontal disease, varies from region to region, due to differences in people's interest in their oral health, differences in dietary habits, and the implementation of fluoridation. In order to accurately clarify the relationship between mandibular protrusion and oral diseases, it is thought that surveys in a large number of regions will be necessary. In addition, this study did not include an examination of relationships with the patient's lifestyle, diet, or general condition. These factors may have affected the data as they are involved in the occurrence of caries and periodontal disease.

## Conclusions

This study investigated decayed, missing, and filled teeth in patients with mandibular protrusion and calculated the DMFT index, tooth survival rate, tooth decay rate, and rate of Filled teeth, Crown teeth, abutment teeth, and root canal-treated teeth by age group. The DMFT indices of patients with mandibular protrusion were higher in all age groups than in the 2016 Survey of Dental Diseases in Japan, raising the possibility that patients with mandibular protrusion may be more susceptible to some oral diseases, such as caries, periodontal disease, and occlusal trauma. Moreover, the condition of the molar region may tend to deteriorate compared to other regions. It is thought that the progression of occlusal collapse in patients with jaw deformity (mandibular protrusion) may start from the molar.
